# A Comparison Between Pressure Wire and Microcatheter Measurements for Evaluating the Cerebral Venous Pressure Gradient

**DOI:** 10.3389/fneur.2021.711870

**Published:** 2021-10-15

**Authors:** Anat Horev, Dana Lorber, Noa Vardi-Dvash, Yair Zlotnik, Ron Biederko, Gal Ifergane, Ilan Shelef, Vladislav Zvenigorodsky, Amir Horev

**Affiliations:** ^1^Neurology Department, Soroka University Medical Center, Beersheba, Israel; ^2^Faculty of Health Sciences, Ben-Gurion University of the Negev, Beersheba, Israel; ^3^Department of Molecular Genetics, Weizmann Institute of Science, Rehovot, Israel; ^4^Clinical Research Center, Soroka University Medical Center, Beersheba, Israel; ^5^Radiology Department, Soroka University Medical Center, Beersheba, Israel; ^6^Pediatric Division, Soroka University Medical Center, Beersheba, Israel

**Keywords:** *Pseudotumor cerebri*, venous stenosis, idiopathic intracranial hypertension, cerebral venous pressure, manometric analyses, pressure wire

## Abstract

**Introduction:** A pressure gradient of over 8 mm Hg across the stenosis (usually located in the transverse-sigmoid junction) is one of the criteria for cerebral venous stenting in idiopathic intracranial hypertension (IIH) patients. The possible inaccuracy of the traditional microcatheter-based pressure measurements has been discussed in previous studies. In the cardiology field, a dual-sensor pressure wire is routinely used for the evaluation of stenotic lesions. Using a pressure wire for cerebral vasculature was previously discussed in a small case series and case reports. In this study, we compared venous pressure measurements obtained using both a microcatheter and a pressure wire in patients who were candidates for stenting.

**Methods:** A retrospective study was conducted, comparing the two methods of pressure measurements in 26 patients with venous stenosis. Altogether, 120 measurements were performed using both methods. Demographic characteristics, medical history, procedural details, medications, indications for the procedure, and complications were collected from the patient charts.

**Results:** Based on an 8-mm Hg pressure gradient cutoff indication, 19 patients were found eligible to go through unilateral venous stenting based on catheter measurements alone. The wire results corroborated the catheter results in detecting all cases indicated for a stent. This finding implies a sensitivity equal to 100% for the wire measurements. There were no wire-related complications, demonstrating its safety.

**Conclusions:** We conclude that the pressure wire is as safe as the microcatheter and can identify cases requiring intervention. A larger-scale study is needed to assess the measurement accuracy of the pressure wire in brain vasculature.

## Introduction

More than 90% of idiopathic intracranial hypertension (IIH) patients are known to have cerebral venous sinus stenosis (1, 2). Based on these findings, venous stenting has become an established treatment option for IIH patients, especially for refractory cases (3, 4). One of the criteria for stenting IIH patients is having a pressure gradient of over 8 mm Hg measured before and after the stenosis (5–8). Traditionally, venous pressure measurements are performed through a 3/4 FR microcatheter attached distally to a standard hydraulic pressure recording system used for vascular pressure monitoring (5–8). Inaccuracy in catheter-based pressure measurements has been discussed in past studies (9–12). Dual-sensor pressure wires have been routinely used for over 20 years in the cardiology field for gradient measurement over stenotic lesions (13). Over the past 10 years, only a few case reports and case series have studied the safety and possible accuracy of using a pressure wire for measurements of brain vasculature (10, 11, 14–16). Since doubts exist regarding the accuracy of catheter-based venous measurement, we decided, in our medical center, to perform both catheter and pressure wire measurements in all cases before making the decision to deploy a stent in the venous system. In this study of 26 IIH patients, we compared cerebral venous measurements gathered using microcatheters to those acquired using pressure wires. Our study tests the safety and accuracy of the pressure wire.

## Methods

In this retrospective analysis, we compared two methods of pressure measurements from the cerebral venous system in patients with venous stenosis. Demographic characteristics, medical history, procedural details, medications, indications for the procedure, and complications were collected from the patient charts. The institutional ethics committee of the Soroka University Medical Center approved the study protocol.

### Study Population

The study population included patients over 18 years old who had undergone elective diagnostic venography and venous pressure measurements at Soroka University Medical Center between January 2015 and October 2020. The indication for the procedure was evaluation and possible venous stenting due to refractory IIH or isolated pulsatile tinnitus (PT). Prior to the procedure, all patients went through a brain MRI/MRV that ruled out malignancy or other pathological findings and showed suspected uni- or bilateral transverse/sigmoid sinus (TSS) stenosis.

### Procedural Management

Before the procedure, the patients were treated with 7 days of 100 mg aspirin and 75 mg of clopidogrel once daily. Cerebral venography was performed under general anesthesia. After transfemoral venous access was obtained, a Navien 072 (Medtronic) inside a 6Fr NeuronMax guide catheter (Penumbra, Alameda, CA, USA) was advanced into the proximal segment of the dominant sigmoid sinus.

The patient was treated with intravenous heparin during the procedure to keep an activated clotting time (ACT) over 250 s. A microcatheter (3MAX, Penumbra) was guided over a 0.14-inch microwire (Synchro-2, Boston Scientific, Marlborough, MA, USA) into the superior sagittal sinus. An injection through the microcatheter was performed to verify and locate the stenotic region(s). After the stenosis was crossed, the microwire was removed, and a standard arterial pressure transducer was calibrated and connected to the microcatheter. Systolic, diastolic, and mean measurements were recorded. The same measurements were also recorded proximal and distal to the stenosis in the contralateral side (in case of demonstrated contralateral stenosis). After the microcatheter pressure measurements were completed, we crossed the stenotic lesion again in the same manner and then exchanged the Synchro wire for a pressure wire (Verrata Philips) and pulled back the microcatheter into the Neuron MAX. The wire was connected to its computerized system, and pressure recordings were collected from the exact locations before and after the stenotic areas.

We ensured that during all measurements, the microcatheter and Navien were pulled back into the Neuron MAX that was always located at the proximal segment of the sigmoid sinus (as far as possible to avoid interference with measurements). In cases where a gradient of ≥8 mm Hg was recorded with the microcatheter (currently the gold standard), we proceeded with stenting. Patients who were not found to have a significant pressure gradient did not go undergo stenting, and the procedure was completed at this point.

### Venous Sinus Stenting

An appropriately sized Precise (Cordis) stent (7–9 mm diameter, 40 mm length) was placed in eligible patients. If bilateral stenosis with a bilateral significant pressure gradient was found, the stent was placed on the dominant side with the higher-pressure gradient. Post-stenting balloon dilatation was done only rarely, with a 7 × 40 Ballon 7 × 40 (Aviator, Cordis), whenever we had the impression that there was a stenotic region within the stent structure. A pressure gradient was remeasured with both a microcatheter and a pressure wire post-stenting, proximal and distal to the stent.

## Results

**Figure 1:**
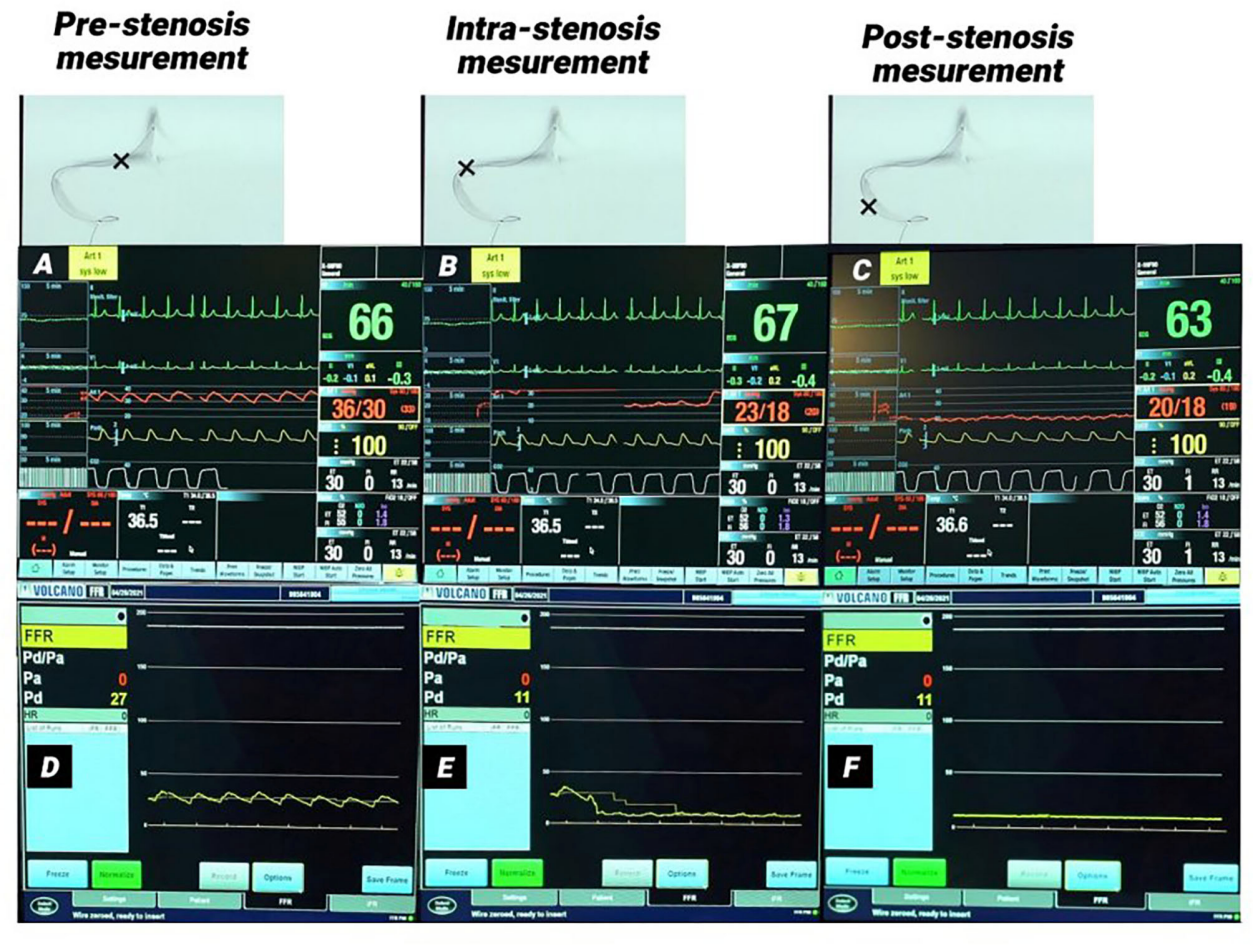
Monitor picture of arterial and pressure wire measurements in a patient with right transverse-sigmoid sinus stenosis. **(A–C)** Monitor pictures of the arterial line measurements pre-stenosis **(A)**, intra-stenosis **(B)**, and post-stenosis **(C)**. **(D–F)** monitor pictures of pressure wire measurements pre-stenosis **(D)**, intra-stenosis **(E)**, and post-stenosis **(F)**. A significant pressure gradient was measured in both methods. **(B,E)** Demonstrate the pressure drop in both measurements at the time the lesion was crossed.

**Figure 2:**
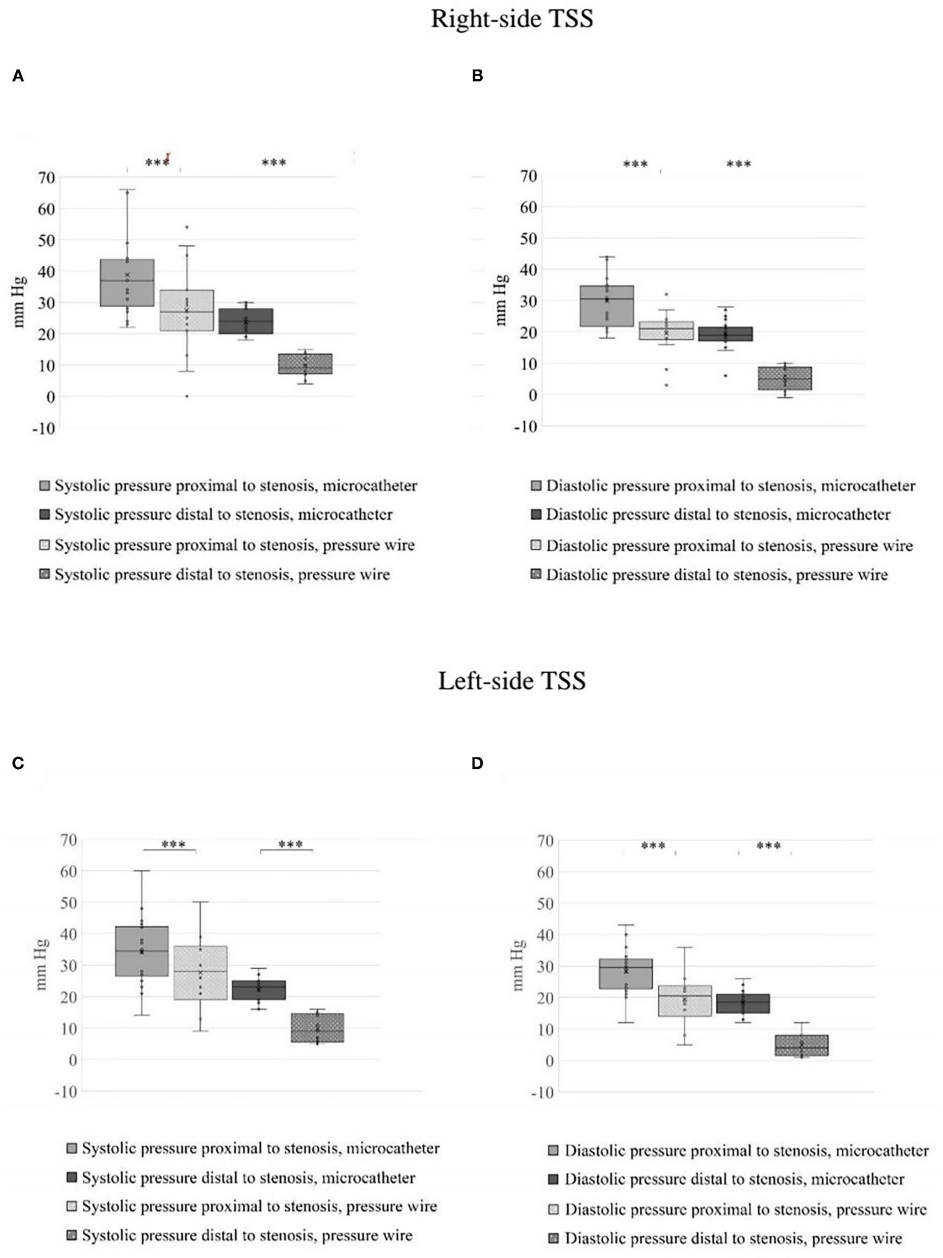
Estimated blood pressure across the stenosis on both sides before treatment. **(A)** Right systolic venous pressure (SVP) and **(B)** right diastolic venous pressure (DVP) across the stenosis obtained by the microcatheter and pressure wire. **(C)** Left SVP and **(D)** left DVP across the stenosis obtained by the two measurements systems. The blood pressure range measured by the two methods, for all the cases tested, differ significantly, ***p < 0.001.

**Figure 3:**
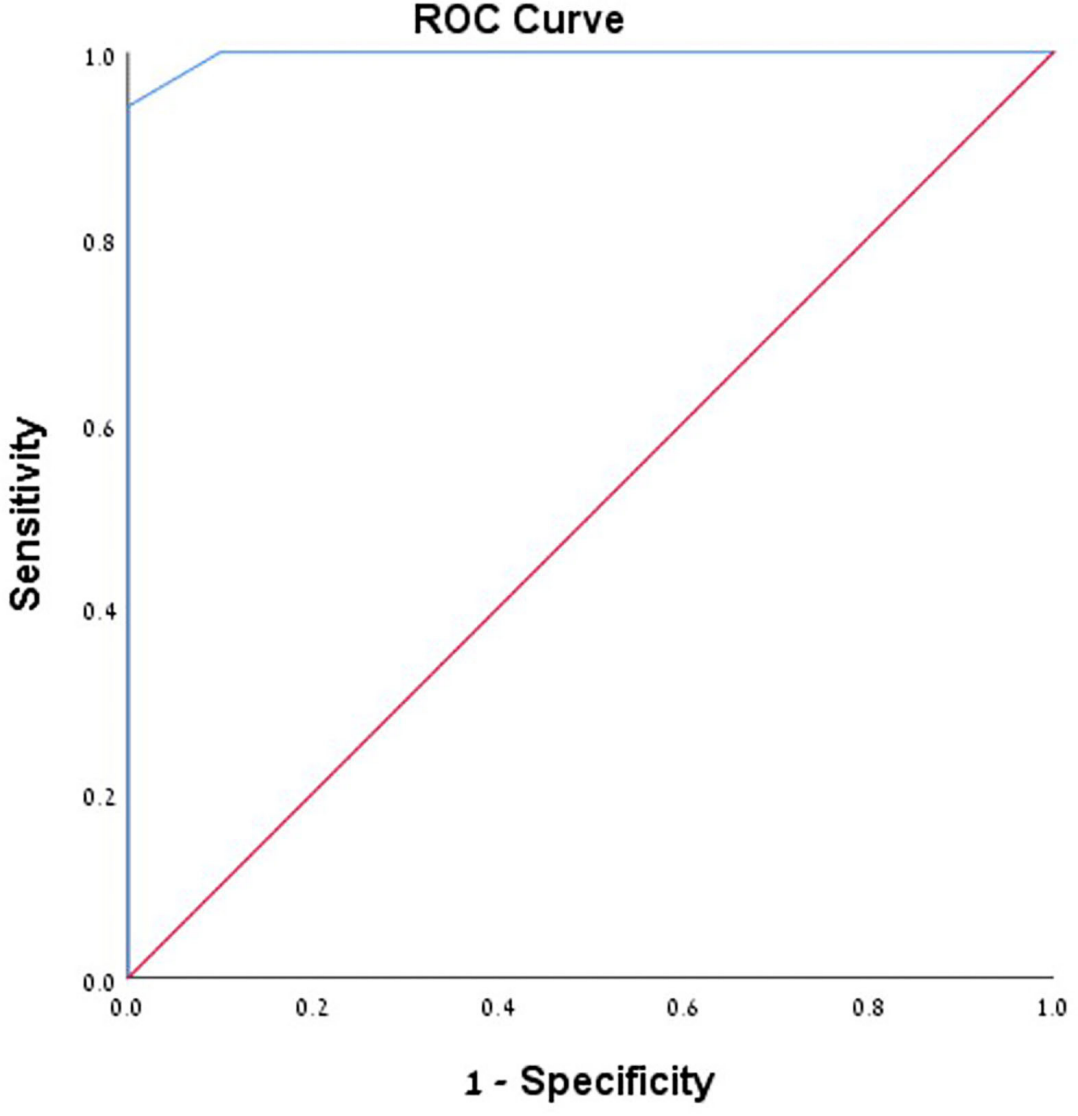
ROC curve analysis of pressure wire method.

**Figure 4:**
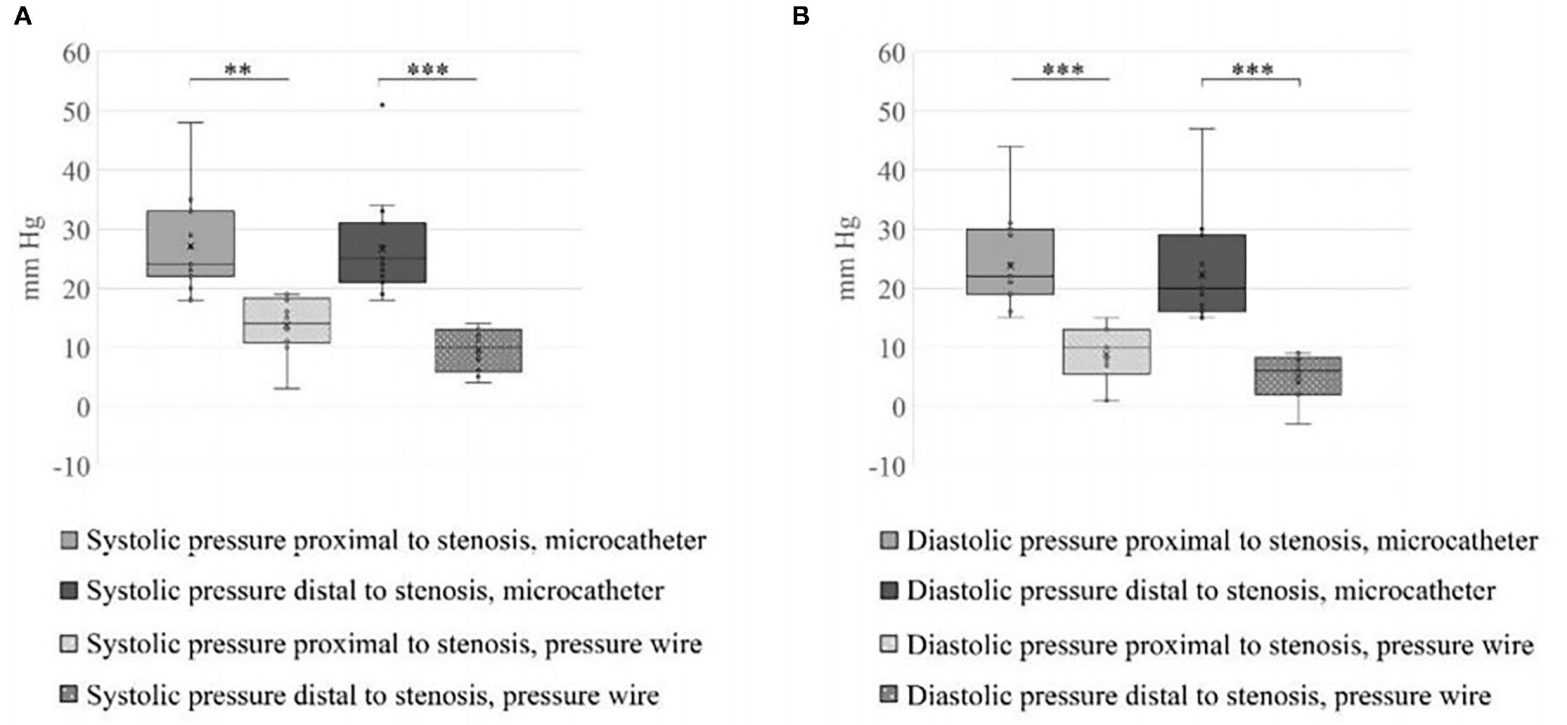
Systolic and diastolic values measured proximal and distal to the stent. **(A)** SVP and **(B)** DVP across the treated stenosis obtained by the two measurements systems. The blood pressure range measured by the two methods, for all the cases tested, differ significantly (**p < 0.01, ***p < 0.001).

A total number of 26 female patients were included in the study. Three of them had an isolated PT, and 23 had been diagnosed with IIH and had either developed severe side effects to medical treatment or showed no significant response to it. The mean age of the patients at the time of the procedure was 34.12 ± 9.88 years (range 18–52). Additional demographic and clinical information is shown in Table 1. A total of 120 paired pressure measurements were recorded by both methods from the 26 patients. All patients underwent pre- and post-stenosis venous pressure measurements with both pressure wire and a microcatheter attached to an arterial line transducer system (Figure 1). In most of the patients, we were able to perform bilateral measurements. Results of the measurements are detailed in Table 2. Measurements were recorded during the procedure, before stenting, on both TSS sides (A: right, B: left), and the calculated values are based on them. A paired Student *t*-test was performed for each parameter measured by the two systems. Microcatheter pressure values were significantly higher than the pressure wire for all measurements, as presented in Figure 2. Venous pressure measurements pre- and post-stenosis as measured by the arterial line and pressure wire differ on both sides. Figure 2 displays the measured right-side systolic (Figure 2A) and diastolic (Figure 2B) pressure and the measured systolic (Figure 2C) and diastolic (Figure 2D) pressure across the left TSS. In all described locations, significantly higher values were measured by the arterial line. Based on an 8-mm Hg pressure gradient cutoff indication, 19 patients were found eligible to go through unilateral venous stenting, based on catheter measurements alone. The wire results corroborated the catheter measurements in detecting all cases indicated for a stent. This finding implies a sensitivity equal to 100% for the wire measurements. Of the patients not in need of stenting per the catheter measurements, three patients theoretically would be assigned for the procedure based on the wire measurements. This can be translated to a specificity index equal to 60%. We conducted receiver operating characteristic (ROC) analysis to estimate the ability of the pressure wire method to discriminate between patients requiring stenting from those who do not. In the ROC curve analysis shown in Figure 3, a stent was indicated for patients with pressure gradients higher than 8 mm Hg in the catheter measurements. The figure presents the estimates of sensitivity and specificity of the method vs. this indication. The area under the curve (AUC) was estimated at 0.997 (*p* < 0.001). Repeating the same pattern, significantly higher values were also recorded in the catheter post-stenting, as shown in Table 3 and Figure 4. The wire pressure gradient measurements corroborated 100% of the catheter measurements post-stenting as well. Measurements in Table 3 were recorded post-stenting, proximal and distal to the stent. A paired Student *t*-test was performed for each parameter measured by the two systems. Figure 4 demonstrates the systolic venous pressure (SVP) in (Figure 4A), and in (Figure 4B), the diastolic venous pressure (DVP), proximal and distal to the treated stenosis obtained by the two measurement systems. The venous pressure range measured by the two methods, for all cases tested, differed significantly. Regarding complications, one patient developed a groin hematoma, but no other complications were reported. We encountered no wire-related technical problems or wire-related complications.

**Table 1:** Patient demographics and clinical history.

***N*** **=** **26**		
**Gender (male)—*****N*** **(%)**		1 (3.8)
**Age, years**		34.12 ± 9.88
Mean ± SD		(18;52)
(min; max)		
**Weight, kg** –		(23) 81.91± 14.34
(*N*) Mean ± SD		(51; 105)
(min; max)		
**Height, cm** –		(21) 161.90 ± 5.90
(*N*) Mean ± SD		(150; 170)
(min; max)		
**BMI**		(24) 31.96 ± 8.32
(*N*) Mean ± SD		(19; 61)
Median (IQR)		
Topamax treatment—*N* (%)		4 (15.4)
Uramox treatment—*N* (%)		20 (76.9)
Symptoms—*N* (%)	Pseudotumor	10 (38.5)
	Tinnitus	5 (19.2)
	Both	9 (34.6)
**Lumbar puncture opening pressure mm/hg**		
(*N*) Mean ± SD		(21) 341.19 ± 123.32
Median (IQR)		320 (250; 400)
Headache—*N* (%)		23 (88.5)
Papilledema—*N* (%)		20 (76.9)
Visual field defects—*N* (%)		17 (65.4)

**Table 2:** Venous pressure measured with the microcatheter and the pressure wire before stent treatment.

		**Pre-stenosis**			**Post-stenosis**			**Pressure gradient**
		**Systole**	**Diastole**	**Mean**	**Systole**	**Diastole**	**Mean**	
**(A) Pre-treatment pressure measurements—right transverse sigmoid junction (mm Hg)**.								
Micro-catheter	Avg ± SD	38.8 ± 13.2	29.9 ± 8.1	33.9 ± 9.6	23.8 ± 3.9	19.1 ± 4.7	20.3 ± 4.8	13.8 ± 8.0
	*N*	20	20	23	21	21	23	25
Pressure wire	Avg ± SD	29.5 ± 12.3	19.6 ± 7.0	24.7 ± 8.4	9.6 ± 3.4	4.9 ± 3.7	9.6 ± 4.9	15.8 ± 7.3
	*N*	14	14	23	12	12	23	25
*t* test	*p value*	<0.001	<0.001	<0.001	<0.001	<0.001	<0.001	0.048
	*N*	13	13	23	12	12	24	25
**(B) Pre-treatment pressure measurements—left transverse sigmoid junction (mm Hg)**								
Micro-catheter	Avg ± SD	34.2 ± 10.7	28.3 ± 7.4	31.8 ± 9.2	22.2 ± 3.8	18.3 ± 3.6	19.8 ± 3.4	12.2 ± 7.8
	*N*	18	18	20	18	18	20	20
Pressure wire	Avg ± SD	27.6 ± 11.5	19.5 ± 8.3	26.7 ± 11.3	9.8 ± 4.1	5.0 ± 3.6	10.9 ± 5.4	15.7 ± 7.9
	*N*	10	10	19	9	9	19	19
*t* test	*p value*	<0.001	<0.001	0.0017	<0.001	<0.001	<0.001	<0.001
	*N*	10	10	19	9	9	19	19

**Table 3:** Venous pressure measured with the microcatheter and the pressure wire post-stenting.

	**Post-stenting pressure measurements (P, mm Hg)**							
		**Pre-stenosis**			**Post-stenosis**			**Pressure gradient**
		**Systole**	**Diastole**	**Mean**	**Systole**	**Diastole**	**Mean**	
Micro-catheter	Avg ± SD	27.1 ± 7.7	23.8 ± 7.5	25.4 ± 7.7	26.6 ± 8.1	22.3 ± 8.4	23.4 ± 8.2	2.3 ± 2.3
	*n*	15	15	17	15	15	17	17
Pressure wire	Avg ± SD	13.7 ± 4.7	8.8 ± 4.5	15.8 ± 7.0	9.5 ± 3.4	5.0 ± 3.6	11.3 ± 6.8	4.5 ± 1.7
	N	10	10	18	10	10	18	18
*t* test	*p value*	0.0016	<0.001	<0.001	<0.001	<0.001	<0.001	0.0038
	*n*	8	8	16	8	8	16	16

## Discussion

In this study, we compared two cerebral venous pressure measurement modalities: the microcatheter system, currently the gold standard, vs. the pressure wire system, which is regularly used only in the cardiology field. As far as we are aware, this is the largest case series reporting the use of pressure wire for cerebral venous measurements, with 120 comparison measurements included. Our study demonstrates the safety of pressure wire use in cerebral veins. Using our technique, there were no wire-related complications. Other studies also support the safety of using a pressure wire in the cerebral venous system (10, 11, 15, 16). We also noticed that the wire was in full agreement with the microcatheter system on patients with pressure gradients equal to or >8 mm Hg, which is currently the formal indication for venous stenting (100% sensitivity). In three cases, the wire detected a significant gradient for stenting that was not detected by the microcatheter. In one of the cases, we repeated the microcatheter measurements a few times but could not obtain persistent results, which could indicate a technical failure of the arterial line system. This could occur from several possible malfunctions, such as accidentally changing the location of the transducer, adding extension tubing, kinking of the tubing, or clotting in the tubing system. In the two other cases, the wire showed results that were slightly over the 8-mm Hg cutoff criteria, and the catheter showed a result slightly lower than that. This difference could possibly be explained by a normal deviation between the different measurement systems. We noticed no anatomical differences in these three cases that could provide a better explanation. Significantly higher mean, diastolic, and systolic pressure measurement values were recorded with the microcatheter relative to the pressure wire. These results disagree with a case series that included 14 patients, which showed that the absolute measured values were actually higher in the wire measurements (although no statistical significance was calculated) (10). This difference in absolute results can be explained by the different microcatheters that were used in the other studies. Lenck et al. used a Prowler Select Plus, which has a 2.3-F distal outer diameter (OD) and a 0.015-inch distal inner diameter (ID). In our study, we used a 3 Max, which has a 3.8-F distal OD with a 0.035-inch distal ID. A larger catheter might measure higher values since it narrows the lumen of the vessel. Other studies, on the other hand, have shown that microcatheters with larger ID will have lower damping (17, 18) since damping is proportional to one-third of the tube radius (19–21). In addition, enlarging the tube diameter will increase the natural frequency of the system, distinguishing it from the blood pressure fluctuation frequency and minimizing the risk of resonance (21, 22). These results suggest using larger ID catheters for pressure measurements. Lengthening the tube may have an adverse effect on the pressure measurements since it decreases the natural frequency of the system (21, 22) and increases damping (19). In addition to catheter dimensions that may influence the measurements, other issues that can affect the waveform are the tubing materials and the vessel curves that may also exhibit artifact-causing attenuation, overshooting, or damping. Another significant difference between the two measurement systems that may have a significant effect on the measured values is the location of the pressure sensor itself. The sensor of the pressure wire is located close to the tip of the wire, whereas the pressure sensor of the microcatheter is at the end of the fluid column, located on the procedural bed, which is more than 100 cm from the stenotic area. The pressure wire method has been used as the gold standard for measurement in cardiology for the past 20 years and is considered highly accurate (13, 23, 24).

Our measurements lead us to conclude that the pressure wire is safe and probably as accurate as the microcatheter. As a more straightforward method without dependence on multiple variables, it is theoretically possible that the pressure wire is even more accurate than the microcatheter. Besides the fact that this study is a retrospective study, another limitation of the study is its small scale (although it is the largest published study discussing the topic, of which we are aware). Since there is currently no absolute way to measure venous pressure, we were unable to prove a higher accuracy of the wire over the catheter in the present study and thus propose larger-scale studies to help investigate this likelihood.

## Data Availability Statement

The raw data supporting the conclusions of this article will be made available by the authors, without undue reservation.

## Ethics Statement

The studies involving human participants were reviewed and approved by Helsinki-Soroka. Written informed consent for participation was not required for this study in accordance with the national legislation and the institutional requirements.

## Author Contributions

AnH and DL conceived the original project idea and produced the manuscript. AnH collected the data. RB and DL ran statistical analyses. NV-D, YZ, GI, IS, VZ, and AmH did literature review and helped AnH and DL to write the article. All authors contributed to the article and approved the submitted version.

## Conflict of Interest

The authors declare that the research was conducted in the absence of any commercial or financial relationships that could be construed as a potential conflict of interest.

## Publisher's Note

All claims expressed in this article are solely those of the authors and do not necessarily represent those of their affiliated organizations, or those of the publisher, the editors and the reviewers. Any product that may be evaluated in this article, or claim that may be made by its manufacturer, is not guaranteed or endorsed by the publisher.
